# Health economic evaluation of rivaroxaban in elective cardioversion of atrial fibrillation

**DOI:** 10.1007/s10198-017-0942-2

**Published:** 2017-11-27

**Authors:** Maartje S. Jacobs, Lisa A. de Jong, Maarten J. Postma, Robert G. Tieleman, Marinus van Hulst

**Affiliations:** 1 Department of Clinical Pharmacy and Toxicology, Martini Hospital, Van Swietenplein 1, 9728 NT Groningen, The Netherlands; 2 Unit of Pharmacotherapy, Pharmacoepidemiology and Pharmacoeconomics, Groningen Research Institute of Pharmacy, University of Groningen, Antonius Deusinglaan 1, 9713 AV Groningen, The Netherlands; 3 Institute for Science in Healthy Aging and Healthcare, University Medical Center Groningen, University of Groningen, Antonius Deusinglaan 1, 9713 AV Groningen, The Netherlands; 4 Department of Epidemiology, University Medical Center Groningen, University of Groningen, Hanzeplein 1, 9713 GZ Groningen, The Netherlands; 5 Department of Cardiology, Martini Hospital, Van Swietenplein 1, 9728 NT Groningen, The Netherlands; 6 Department of Cardiology, University Medical Center Groningen, University of Groningen, Hanzeplein 1, 9713 GZ Groningen, The Netherlands

**Keywords:** Cardioversion, Oral anticoagulation, Rivaroxaban, Vitamin K oral antagonists, Health economic evaluation, I10 Health, General, C63 Computational Techniques, Simulation Modeling, D61 Allocative Efficiency, Cost-Benefit Analysis

## Abstract

**Background:**

Electrical cardioversion (ECV) is a procedure in which a direct current electric shock is used to quickly and effectively restore the normal sinus rhythm. Appropriate anticoagulation reduces the risk of embolic events during and after ECV. The aim of this study was to estimate the cost-effectiveness of rivaroxaban compared with vitamin K oral antagonists (VKAs) in patients with atrial fibrillation undergoing elective ECV in the Netherlands.

**Methods and results:**

A static transmission model over a 1-year time horizon was developed to compare rivaroxaban with VKAs in terms of clinical outcomes, health effects (quality-adjusted life years; QALYs), and costs. Cost-effectiveness was assessed from a societal and health care payer perspective at a willingness-to-pay level of €20,000 per QALY gained. The use of rivaroxaban as an anticoagulant in patients with atrial fibrillation scheduled for ECV would lead to a health gain of 0.23 QALYs per patient and would cost €1.83 per patient from the societal perspective, resulting in an incremental cost-effectiveness ratio of €7.92 per QALY gained. The probability of rivaroxaban being cost-saving compared with VKAs was 49.6% from this perspective. From the health care payer perspective, the incremental cost would be €509 per patient with a health gain of 0.23 QALYs per patient, resulting in an incremental cost-effectiveness ratio of €2198 per QALY gained.

**Conclusions:**

The use of rivaroxaban in elective ECV is a cost-effective alternative to the use of VKAs. Rivaroxaban has a 50% probability of being cost-saving compared with VKAs and would increase a patient’s quality of life when non-health care costs such as productivity loss and informal care costs are taken into account.

**Electronic supplementary material:**

The online version of this article (10.1007/s10198-017-0942-2) contains supplementary material, which is available to authorized users.

## Introduction

Atrial fibrillation (AF) is a cardiac arrhythmia with a prevalence of 5.5% in the population older than 55 years in the Netherlands, and prevalence increases with age [1, 2]. In the Netherlands, AF is newly diagnosed in around 45,000 patients every year, and the total cost for all AF patients (including hospitalization, in-hospital procedures, and pharmacotherapeutic management) has been estimated at €600 million per year [2]. Patients with AF have a fivefold increased risk of stroke and increased chance of developing systemic embolism (SE) [3]. Anticoagulants can be used to reduce the thromboembolic risk. Symptomatic AF patients have a lower quality of life compared with nonsymptomatic AF patients. Symptoms can be very limiting for a patient, ranging from “troubled by symptoms but normal daily activity not affected” up to the point where normal daily activity is discontinued [4]. Severe and disabling AF, which accounts for around 30% of all AF patients, can have a great economic impact because of a decrease in general health and subsequent loss of work productivity [5]. The thromboembolic risk appears not to be affected by the symptomatic status of an AF patient, and the clinical status should therefore not determine the stroke prevention approach [6]. The symptoms nonetheless do determine the eligibility for cardioversion. In early stages of AF, symptomatic patients can undergo electrical cardioversion (ECV). ECV is a procedure in which a direct current electric shock is used to quickly and effectively restore the normal sinus rhythm [7, 8]. The risk of SE and stroke is increased around the time of ECV, mainly because of a temporary stasis of the blood. Appropriate anticoagulation reduces the risk of these embolic events during and after the ECV procedure, but it increases the risk of bleeding [9]. An elective ECV strategy with at least 3 weeks of anticoagulant treatment is chosen if AF is present for more than 48 h. In elective ECV, the patient has to receive adequate anticoagulation for at least 3 weeks before and at least 4 weeks after the procedure [7]. Patients at risk of stroke are eligible for life-long anticoagulation and can be identified by the CHA_2_DS_2_-VASc score [10]. Nowadays, in patients with AF there is a preference toward prescribing a non-vitamin K antagonist oral anticoagulant (NOAC) over a vitamin K oral antagonist (VKA) [8, 11]. VKAs are relatively inexpensive drugs but have an unpredictable therapeutic response. For this reason, strict monitoring of the patient’s blood coagulation by means of the international normalized ratio (INR) is required. The NOAC rivaroxaban is a direct factor Xa oral antagonist, indicated for prevention of stroke and SE in nonvalvular AF patients. Clinical trials have shown similar effects for rivaroxaban and VKAs in the prevention of stroke and SE in AF patients, and also in those undergoing ECV as shown in the X-VerT trial [12, 13]. Rivaroxaban has a more predictable pharmacokinetic and pharmacodynamic profile and has fewer drug–drug interactions, and INR measurement is not required [12]. High rates of inadequate anticoagulation, up to 50%, have been an extensive problem with VKA use and the most frequent reason to postpone ECV [14–16]. Because of the rapid onset of anticoagulation with rivaroxaban, cardioversion can be performed significantly earlier and inadequate anticoagulation before cardioversion is less frequent [13]. In a post hoc budget-impact analysis of the X-VeRT trial, it was estimated that the use of rivaroxaban compared with a VKA resulted in a cost saving of £421 per patient in the UK setting and €360 per patient in the Italian setting [14]. This budget-impact analysis did not include AF-related event costs and non-health care costs. An extensive cost-effectiveness analysis including the total budget impact of all costs relevant for elective ECV with rivaroxaban has not yet been published. The aim of this study was to estimate the cost-effectiveness of rivaroxaban compared with a VKA in patients with AF undergoing elective ECV in the Netherlands.

## Methods

### Overview

In this health economic evaluation, we used a static transmission model over a 1-year time horizon to compare rivaroxaban with VKAs in elective ECV in terms of clinical outcomes, health effects, and costs. The clinical outcomes were AF-related events, such as stroke and hemorrhage. Costs were assessed from a societal perspective, also accounting for non-health care costs due to productivity losses and informal care, which is in line with the Dutch pharmacoeconomic guidelines [17]. The final outcomes were incremental costs and quality-adjusted life years (QALYs). Cumulative health effects, direct costs, and indirect costs were determined for elective ECV with rivaroxaban and VKAs. The incremental cost-effectiveness ratio (ICER) of rivaroxaban compared with VKAs was calculated by division of the difference in costs by the difference in QALYs, Rivaroxaban was considered cost-effective at a conservative willingness-to-pay (WTP) level of €20,000 per QALY gained. Costs and health effects were not discounted since the time horizon of the model was limited to 1 year.

### Model design

A decision-analytic model was developed in Excel 2010 to describe the ECV procedure, distinguishing the pre-ECV and post-ECV periods. A hypothetical cohort of 10,000 patients with newly diagnosed symptomatic AF was followed in 1-day cycles before ECV and 1-week cycles after ECV. The patients were scheduled for ECV when they had a modified European Heart Rhythm Association (mEHRA) classification of 2b (moderate), 3 (severe), or 4 (disabling) [4]. Population characteristics and model assumptions are summarized in Table 1. The data used in the model were extracted from the literature, and access to the original data was not required for the analysis.

**Table 1:** Population characteristics of the hypothetical patient cohort and model assumptions of the Markov model (based on the X-VeRT and XANTUS trials)

	Rivaroxaban	Vitamin K antagonist	Reference
Age (years)	64.4	64.4	[13]
Male sex (%)	74.0	74.0	[13]
CHADS_2_ score	2.0	2.0	[18]
CHA_2_DS_2_-VASc score	3.4	3.4	[18]
CHA_2_DS_2_-VASc score < 1	2.6%	2.6%	[18]
CHA_2_DS_2_-VASc score < 2	12.7%	12.7%	[18]
Pre-ECV OAC days	22	30	[13]
Target INR	NA	2.5 (2.0–3.0)	[13]
ECV success rate (%)	86.40	86.40	[13]
Inadequate OAC 1st ECV (%)	0.24	44.19	[13]
Inadequate OAC 2nd ECV (%)	0.24	20.00^a^	[13], assumption

*CHADS*_*2*_ congestive heart failure, hypertension, age 75 years or older, diabetes mellitus, prior stroke, transient ischemic attack, or thromboembolism (doubled), *CHA*_*2*_ *DS*_*2*_ *-VASc* congestive heart failure, hypertension, age 75 years or older (doubled), diabetes, prior stroke, transient ischemic attack, or thromboembolism (doubled), vascular disease, age 65–74 years, and sex (female), *ECV* electrical cardioversion, *INR* international normalized ratio, *NA* not applicable, *OAC* oral anticoagulation

^a^Based on a time in the therapeutic range of 60%

**Fig. 1:**
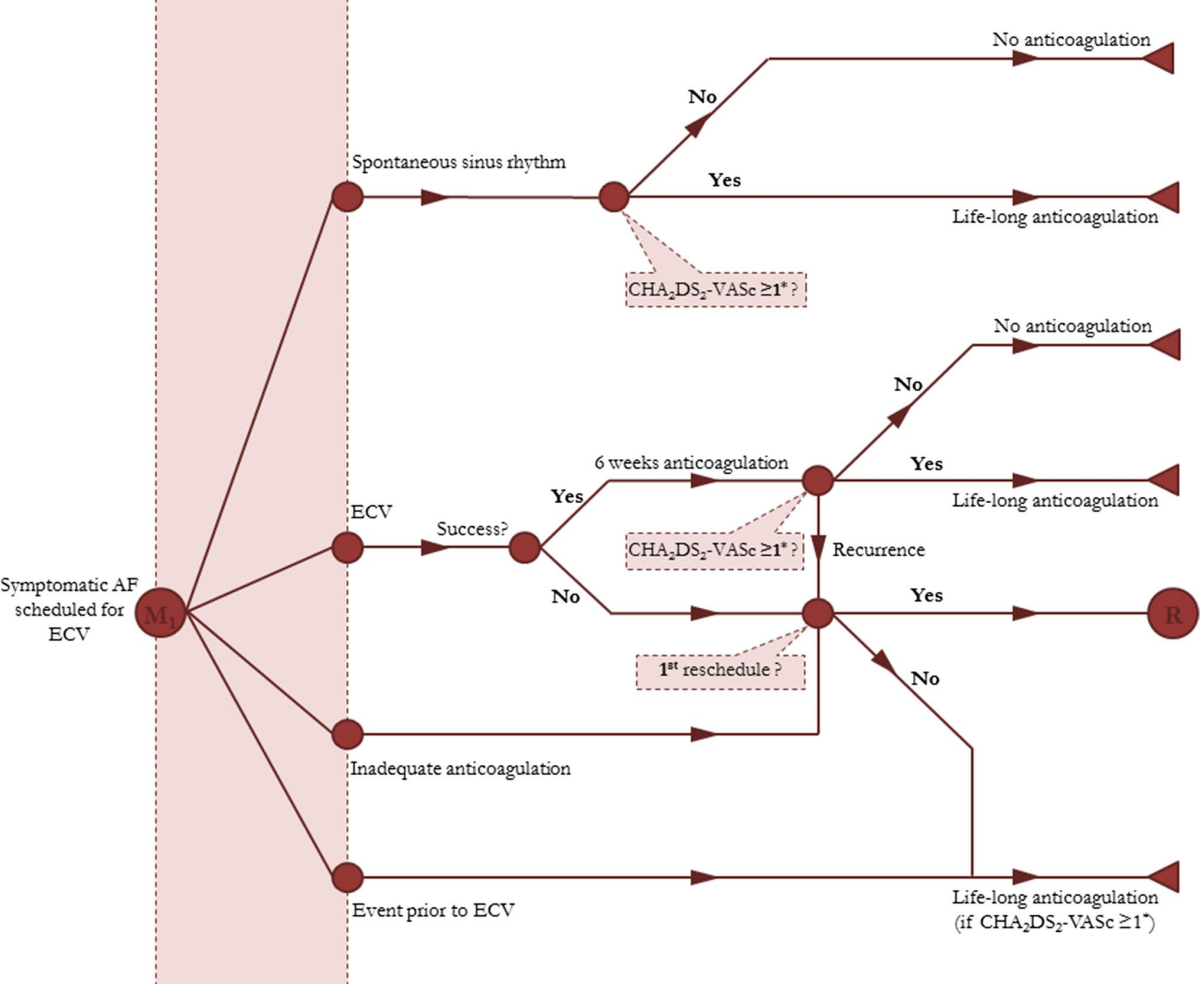
The decision-analytic model. Patients with a first reschedule could reenter (R) the model and would directly start their anticoagulation period before electrical cardioversion (ECV). The red bar indicates the anticoagulation period before ECV, which was different for the base case: 30 days for a vitamin K oral antagonist and 22 days for rivaroxaban. AF atrial fibrillation, CHA_2_DS_2_-VASc congestive heart failure, hypertension, age 75 years or older (doubled), diabetes, prior stroke, transient ischemic attack, or thromboembolism (doubled), vascular disease, age 65–74 years, and sex (female), M1 Markov 1, asterisk CHA_2_DS_2_-VASc score 1 or greater for men and 2 or greater for women

Patients who experienced an event before ECV, except for minor hemorrhage, and patients who were inadequately anticoagulated would not undergo ECV. Patients with inadequate anticoagulation were directly rescheduled for a second ECV. All patients could have an ECV procedure only twice within the time horizon of the model. Patients who experienced an event before ECV or patients with two unsuccessful ECV procedures were categorized as having “permanent AF,” and life-long rate control was initiated. Patients had to continue with oral anticoagulation therapy after ECV for 6 weeks, in accordance with the X-VeRT trial, irrespective of their stroke risk. After this period, men with a CHA_2_DS_2_-VASc score of 1 or greater and women with a CHA_2_DS_2_-VASc score of 2 or greater continued taking the anticoagulant they were already using (rivaroxaban or VKA). Patients who experienced an intracranial hemorrhage (ICH) discontinued anticoagulation therapy. All patients were assumed to start anticoagulation therapy when scheduled for ECV. The model outline is described in Fig. 1.

### Health states and model input variables

**Fig. 2:**
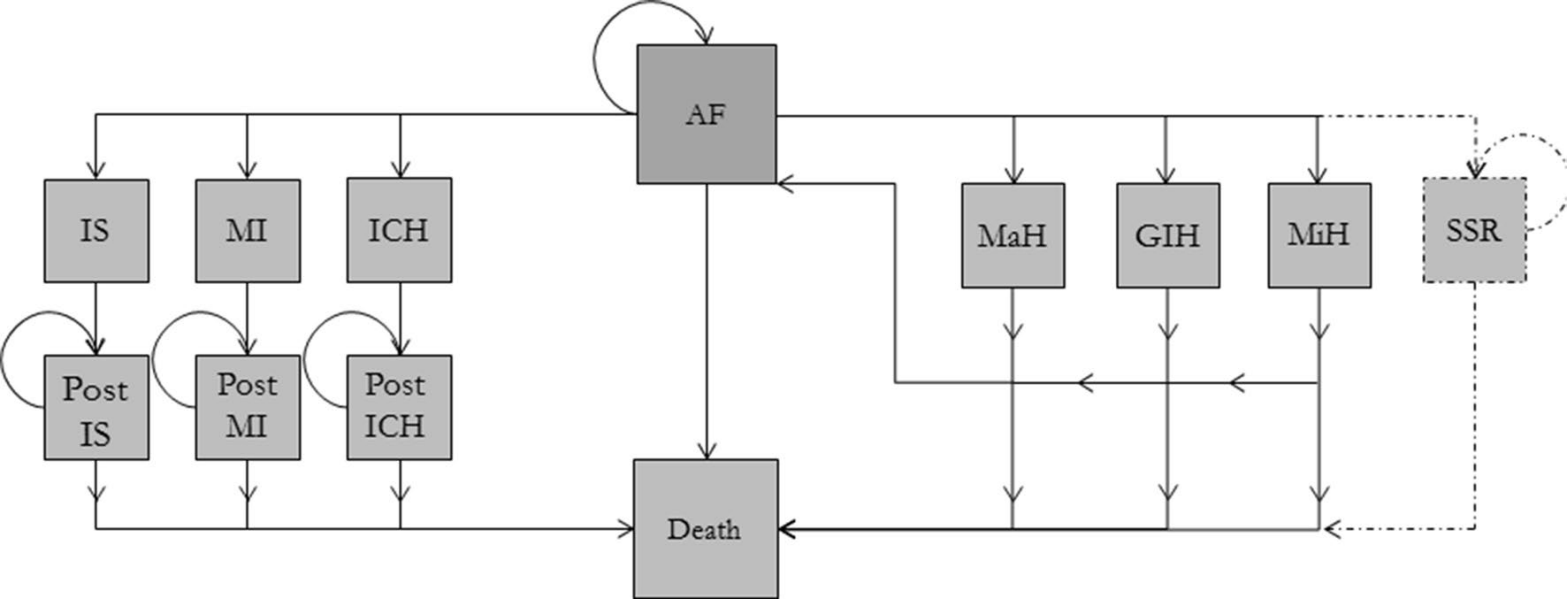
The health states and transition probabilities of the decision-analytic model. The transition probabilities before electrical cardioversion (ECV) for the major hemorrhage (MaH) and gastrointestinal hemorrhage (GIH) states are different from those after ECV. Before ECV, MaH and GIH are absorbing states, and patients experiencing one of these events are excluded from the ECV procedure [same representation as ischemic stroke (IS), myocardial infarction (MI), or intracranial hemorrhage (ICH)]. After ECV, patients will flow back to the atrial fibrillation (AF) state and thus can have multiple bleeding events. The AF state can represent asymptomatic, symptomatic, permanent, or recurrent AF. MiH minor hemorrhage, SSR spontaneous sinus rhythm

All model input variables and their references are listed in Table S1. The health states included within the Markov model are presented in Fig. 2. A transition between these health states can occur at any time point, before ECV and after ECV, and these health states are incorporated in the model structure shown in Fig. 1. Spontaneous sinus rhythm (SSR) can occur at any time point up to the time of the ECV procedure. Major hemorrhage and gastrointestinal hemorrhage states were considered absorbing states before ECV. The event rates were derived from the real-world XANTUS study [18]. The transition probabilities were assumed to be equal in the rivaroxaban and VKA groups to reflect the minimum achievable health gains. The mortality rate for the simulated population was adjusted for age by our increasing the age-specific mortality rate during a patient’s lifetime starting at 64 years [13, 19].

### Health effects

Most of the baseline patient utilities for the specific events were calculated on the basis of EQ-5D scores for the International Classification of Diseases ninth revision (ICD-9) codes. The weighted average utility for symptomatic AF was based on the mEHRA class 2b, 3 and 4 distribution and the utilities matching these classes [4]. The utility of asymptomatic AF was the weighted average of mEHRA classes 1 and 2a. The utility of permanent AF was the weighted average of all mEHRA classes, assuming that rate control would reduce the share of symptomatic patients. Utilities for ischemic stroke (IS) and ICH were based on a previously published nonrandomized cluster trial that explored the severity of IS and ICH and associated costs (see Tables S2 and S3 for the calculations and references). Minor hemorrhage (MiH) had no disutility in our model.

### Costs

All costs (in euros) were updated to the year 2015 by application of a correction for inflation. Drug costs were based on total costs as presented by the Dutch Care Institute. INR monitoring costs were an average of the costs of home monitoring (43.1%) and the regular thrombosis service (56.9%). INR monitoring costs before cardioversion were calculated on the basis of 5.5 measurements on average before the ECV procedure. Periodic monitoring was on average 21.1 and 23.5 measurements per year in patients using the thrombosis service or home monitoring, respectively. INR monitoring data were based on reports from the Federation of Dutch Thrombosis Services. The same underlying calculation based on the severity and duration of the event as mentioned for the utilities was applied for the costs for the IS and ICH states. The cost for myocardial infarction (MI) is the mean weighted treatment cost for acute and postevent costs, with no differentiation for the type of MI and the type of intervention applied. Costs for minor hemorrhage were based on one emergency department visit, and the costs for major hemorrhage were based on the treatment costs for a gastrointestinal hemorrhage. The costs for an ECV were based on the average all-in declaration tariff, expert opinion, and in-house experience. A “last minute” cancellation (canceled 48 h or less before the planned intervention) was calculated as half of the full tariff. The cost of cancellations more than 48 h before the planned intervention was calculated as the administrative costs only, represented by 30 min of work of a specialized nurse. The cost of an ECV cancellation because of SSR was considered as half the full tariff plus an extra policlinic consult with a cardiologist to determine the further treatment policy. Productivity loss was calculated into a single value based on the average population age of 64 years (standard deviation of 10.8 years), the ratio of men to women, the labor market participation per 5-year age group, the average hours of work per week per 5-year age category, and the average monthly gross salary per 5-year age category. A detailed explanation of the calculations can be found in Table S4. The costs of informal care were subdivided into “nonintensive” with 8 h of care provided per week and “intensive” with on average 26 h of care provided per week. Nonintensive informal care was considered for symptomatic AF patients, permanent AF patients, and patients who experienced a major hemorrhage or gastrointestinal hemorrhage. Intensive informal care was considered for the major events of IS, MI, and ICH during the remaining time horizon. The non-health care costs, both productivity loss and informal care costs, were applied to all symptomatic patients in mEHRA classes 3 and 4 during the remaining time horizon. The symptomatic patients in mEHRA classes 3 and 4 reflect 70% of all AF patients before ECV and 32% of all AF patients after ECV.

### Sensitivity analyses

Probabilistic sensitivity analyses (PSA) were performed to assess the robustness of the results. The model input parameters were varied within their 95% confidence intervals where possible, otherwise a standard error of 10–20% was assumed. Drug costs and indirect costs were considered at fixed prices. Event probabilities and utilities were assumed to have *β* distributions; costs were assumed to have *γ* distributions. A total of 10,000 iterations were performed for each combination of parameters, and the results were plotted in a cost-effectiveness plane. The effect of non-health care costs was also assessed with a PSA limited to the health care payer perspective (i.e., omitting productivity loss costs and informal care costs). A series of univariate analyses were performed to explore the effect of key parameter inputs by ranging them over alternative plausible ranges. Age, sex, and inadequate anticoagulation for VKAs were explored over a range of -20% to +20% of the mean deterministic value. To explore the effect of nonadherence to rivaroxaban therapy, the percentage of inadequately anticoagulated patients before ECV in the rivaroxaban group was considered to be equal to the percentage in the VKA group (44.19%) as reported in the X-VeRT trial [13]. The effects of ECV costs (postponed early, postponed at the last minute, and postponed because of SSR) and non-health care costs were assessed by our taking 50% of the mean as the lower value and 200% of the mean as the upper value. Alternative treatment periods with regard to the ECV procedure were considered for rivaroxaban. For the lower value, a period of 12 days of anticoagulation before ECV was considered. The anticoagulation periods before ECV were assumed to be equal (i.e., 30 days for both a VKA and rivaroxaban) for the upper value. The 30-day period is also recommended in the AF guidelines of the European Society of Cardiology [7].

## Results

### Deterministic analysis and probabilistic sensitivity analyses

The use of rivaroxaban as an anticoagulant in patients with AF scheduled for ECV would lead to a health gain of 0.23 QALYs per patient and would cost €1.83 per patient with an ICER of €7.92 per QALY gained compared with the use of VKAs. Around 30–40% of the total cost was allocated to productivity losses, and informal care costs were around 20% of the total cost. The total cost would be €509 per patient, with a health gain of 0.23 QALY per patient from the health care payer perspective and an ICER of €2198 per QALY gained. The results are summarized in Table 2.

**Table 2:** Health effects and total health care costs per patient and the calculated incremental cost-effectiveness ratio (ICER) from the societal and health care payer perspectives

	Cost per patient (€)	QALYs	Incremental cost per patient (€)	Incremental QALYs per patient	ICER (€)
Societal perspective					
Rivaroxaban	4265	0.82	1.83	0.23	7.92
VKAs	4263	0.59			
Health care payer perspective					
Rivaroxaban	2475	0.82	509	0.23	2198
VKAs	1966	0.59			

The ICER calculation was performed with the original, non-rounded numbers and therefore has a very small deviation compared to the division of the rounded incremental values

*QALY* quality-adjusted life year, *VKA*, vitamin K oral antagonist

**Fig. 3:**
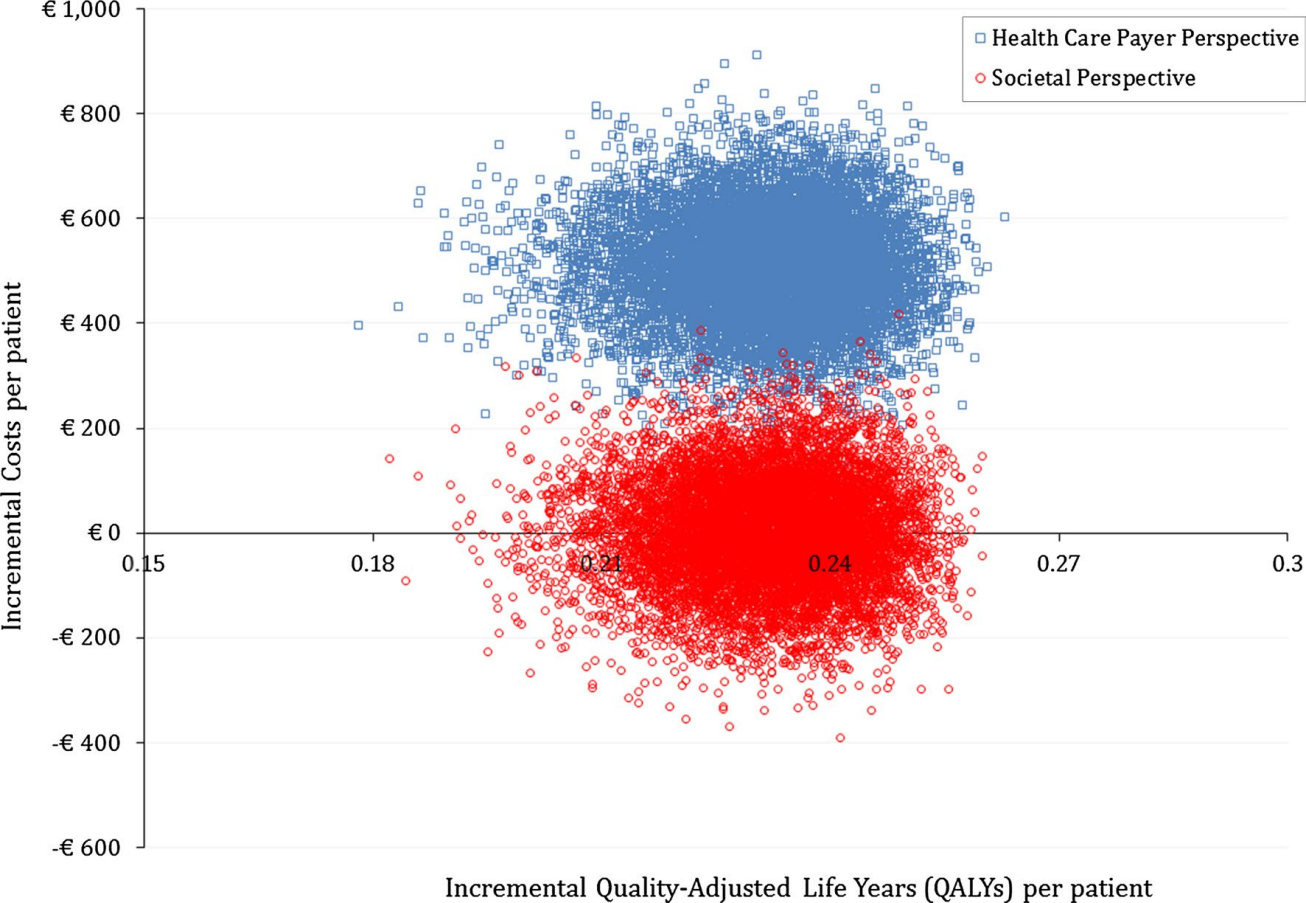
Incremental cost-effectiveness plane showing 10,000 Monte Carlo estimates of incremental health effects (quality-adjusted life years) and cost per patient of elective electrical cardioversion with rivaroxaban versus a vitamin K oral antagonist as an anticoagulant from the societal perspective (circles) and the health care payer perspective (squares)

The PSA from the societal perspective showed that rivaroxaban had a 100% probability of being cost-effective at a WTP level of €20,000 per QALY and would have a 49.6% probability of being cost-saving compared with a VKA. The PSA from the health care payer perspective showed that rivaroxaban had a 100% probability of being cost-effective and would have a 0% probability of being cost-saving compared with a VKA. The PSA results from the societal perspective are plotted in a cost-effectiveness plane in Fig. 3.

### Sensitivity analyses

**Fig. 4:**
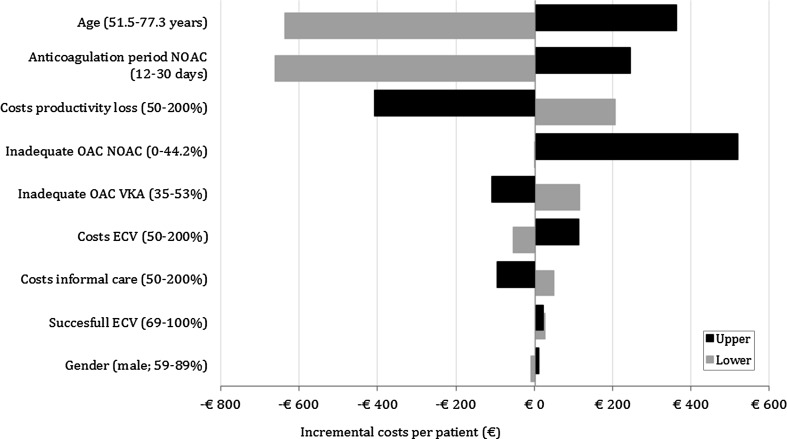
Tornado diagram representing incremental total cost per patient in the univariate analyses. ECV electrical cardioversion, NOAC non-vitamin K antagonist oral anticoagulant, OAC oral anticoagulation, VKA vitamin K oral antagonist

The results of the sensitivity analyses are plotted in a tornado diagram in Fig. 4. Varying sex (the proportion of male patients), the percentage of successful ECV procedures, and the costs of ECV had only a marginal influence on the health effects and costs. The success rate of ECV increased the incremental costs compared with the deterministic scenario in both the lower and the upper range. The impact of age was the greatest in all univariate analyses, with a higher average age of 77.3 years leading to an increase in costs of €360 per patient and a marginal QALY loss of 0.003 per patient compared with an average age of 64.4 years. All other scenarios had a gain in QALYs, except for the univariate analysis with inadequate anticoagulation of 44.19% for rivaroxaban, which resulted in a loss of 0.0015 QALYs per patient. All results are summarized in Fig. 4. Equal anticoagulation periods for VKAs and rivaroxaban before ECV (i.e., both 30 days) would result in a cost increase of €246 per patient and a health gain of 0.24 QALYs per patient compared with VKAs and would still be considered cost-effective at a WTP level of €20,000 per QALY gained. A shorter treatment period would result in higher cost savings. The effect of costs due to productivity loss was substantial, with higher costs resulting in a cost-saving strategy.

## Discussion

In this health economic evaluation we compared the health effect and economic impact of the use of rivaroxaban with use of a VKA before ECV. The use of rivaroxaban was associated with a cost of €1.83 per patient and a health gain of 0.23 QALYs per patient. Choosing rivaroxaban over a VKA would have a 49.6% probability of being cost-saving from a societal perspective and 100% cost-effective at a WTP threshold of €20,000 per QALY gained. From the health care payer perspective, the probability of being cost-saving was 0%. The cost savings are mainly driven by the non-health care costs. The effect of costs due to productivity loss was larger than the effect of costs for informal care. Around 30–40% of the total cost was allocated to productivity losses, and informal care costs were around 20% of the total cost. The robustness of the model was demonstrated in the PSA, and the influence of several key parameters was assessed with multiple sensitivity analyses. The results were most sensitive to the variation in age, the percentage of inadequate anticoagulation, and costs due to productivity losses. For an equal treatment period before ECV but a different percentage of inadequate anticoagulation, rivaroxaban would still be cost-effective, with an ICER of €1010 per QALY gained. This health economic model is, to the best of our knowledge, the first extensive evaluation taking into account all relevant health effects and costs associated with the use of rivaroxaban versus a VKA in elective ECV. A post hoc budget-impact analysis of the X-VeRT trial showed that the use of rivaroxaban in place of warfarin could result in a cost saving of £421 per patient in the UK and €360 per patient in Italy [20]. This post hoc analysis focused only on the direct costs associated with ECV. Patients treated with rivaroxaban in the X-VeRT trial had significantly higher convenience, effectiveness, and global satisfaction scores on the Treatment Satisfaction Questionnaire for Medication version II scale compared with warfarin-treated patients [20].

The effect of “in sinus rhythm” on quality of life has been reported in various studies, mostly based on the 36-Item Short Form Heath Survey (SF-36), although results are contradictory [5, 20]. Variables relevant to the elective ECV procedure and clinical outcomes were derived from the X-VeRT trial and XANTUS study [13, 18]. The use of event rates from a real-world, prospective observational study empowers the external validity of the health economic results. Transition probabilities were assumed to be equal for rivaroxaban and the VKA. The net clinical benefit ratio (the composite of stroke, non-central nervous system SE, transient ischemic attack, MI, cardiovascular death, and major bleeding) was not different between rivaroxaban and VKAs in the X-VeRT trial. Moreover, a subanalysis of the ROCKET-AF trial showed that the incidence of stroke or SE was not different in the rivaroxaban-treated and warfarin-treated patients who underwent ECV, pharmacological cardioversion, or ablation [21]. The impact of the treatment effect is minimal with the short time horizon of the analysis, and therefore the assumption of equal event rates would be reasonable. This 1-year time horizon was chosen so as to focus on the ECV procedure. Using equal event rates makes the approach conservative and therefore represents a minimum achievable health gain. We presume that the results will be comparable for the other NOACs since all of these agents have a direct effect, whereby the number of postponements and/or cancellations would decrease and early cardioversion would be possible. The percentage of inadequate anticoagulation for VKAs has a large impact on the results, influencing the ECV procedure costs and also productivity loss costs since patients will be in a symptomatic state for longer. The X-VeRT trial reported 44.2% inadequate anticoagulation for VKAs (target INR 2.0–3.0 maintained for three consecutive weeks). Although the proportion of inadequately anticoagulated patients seems fairly high, it is representative for the time in therapeutic range during the initiation period of VKA use. Other studies have also reported high, comparable proportions of inadequate anticoagulation before ECV, and this was the primary reason to postpone ECV [14–16]. Nonadherence in the post-ECV period was indirectly covered by use of the real-world event rates from the XANTUS study. These event rates reflect an average treatment effect based on adherent and nonadherent users, and therefore the health economic model developed and its results are a realistic representation of the real-world clinical setting. A 1-year time horizon was chosen in this analysis so as to focus on the ECV procedure and therefore not make it a comparison of long-term anticoagulation. The problem surrounding ECV is, on the one hand, the cost of the high number of cancellations and, on the other hand, the poor quality of life of symptomatic patients who have to wait for their ECV. Our results can support decision making by health care providers and payers since choosing rivaroxaban over a VKA in elective ECV is cost-effective and also improves the planning of the procedure since (last minute) cancellation is less of a problem and relief of patients’ symptoms is accomplished earlier.

## Conclusion

The use of rivaroxaban in elective ECV is considered a cost-effective alternative to the use of VKAs at a WTP level of €20,000 per QALY gained. Rivaroxaban has a 50% probability of being cost-saving and would increase a patient’s quality of life when non-health care costs such as productivity loss and informal care costs are taken into account.

## Electronic supplementary material

Below is the link to the electronic supplementary material.

Supplementary material 1 (DOCX 53 kb)
