# Supplementary material for: Health economic evaluation of rivaroxaban in elective cardioversion of atrial fibrillation
Source: Eur J Health Econ. 2017 Nov 27;19(7):957–65. doi: 10.1007/s10198-017-0942-2 (PMC6105209; doi:10.1007/s10198-017-0942-2)
Supplement: Supplementary file 1 — Supplementary material 1 (DOCX 53 kb) [file 10198_2017_942_MOESM1_ESM.docx]

SUPPLEMENTAL MATERIAL

Health economic evaluation of rivaroxaban in elective cardioversion for atrial fibrillation

European Journal of Health Economics

Maartje S. Jacobs^a b *^, Lisa A. de Jong^b *^, Maarten J. Postma^b c d^, Robert G. Tieleman^e f^, Marinus van Hulst^a b^

* Both authors contributed equally to the work published

1. Department of Clinical Pharmacy and Toxicology, Martini Hospital, Van Swietenplein 1, 9728 NT Groningen, The Netherlands
2. University of Groningen, Groningen Research Institute of Pharmacy, Unit of PharmacoTherapy, -Epidemiology & -Economics (PTEE), Antonius Deusinglaan 1, 9713 AV Groningen, The Netherlands
3. University of Groningen, Institute for Science in Healthy Aging & healthcaRE (SHARE), University Medical Center Groningen (UMCG), Antonius Deusinglaan 1, 9713 AV Groningen, The Netherlands
4. University of Groningen, Department of Epidemiology, University Medical Center Groningen (UMCG), Hanzeplein 1, 9713 GZ Groningen, The Netherlands
5. Department of Cardiology, Martini Hospital, Van Swietenplein 1, 9728 NT Groningen, The Netherlands
6. University of Groningen, Department of Cardiology, University Medical Center Groningen, Hanzeplein 1, 9713 GZ, Groningen, The Netherlands

**Corresponding author:** M.S. Jacobs, m.jacobs@mzh.nl

**Model input variables**

Table S1. Model input variables; event probabilities, costs and utility estimates and their 95% confidence interval.

| **Variable** | **Event probability (week)** | **95% CI estimate** | | | **Reference** |
| --- | --- | --- | --- | --- | --- |
|  |  | **Lower** | | **Upper** |  |
| **Events** | | | | | |
| Spontaneous Sinus Rhythm (SSR) | 2.3299E-02 |  | |  | (1) |
| Ischemic Stroke | 1.3461E-04 | 8.1841E-05 | | 1.8737E-04 | (2) |
| Intracranial Hemorrhage | 7.6920E-05 | 4.6767E-05 | | 1.0707E-04 | (2) |
| Myocardial Infarction | 7.6920E-05 | 4.6767E-05 | | 1.0707E-04 | (2) |
| Major Hemorrhage | 1.3461E-04 | 8.1841E-05 | | 1.8737E-04 | (2) |
| Gastrointestinal Hemorrhage | 1.7306E-04 | 1.0522E-04 | | 2.4090E-04 | (2) |
| Minor Hemorrhage | 2.9572E-03 | 1.7980E-03 | | 4.1164E-03 | (2) |
|  |  |  | |  |  |
|  | **Mean Costs (€)** | **95% CI estimate** | | |  |
|  |  | **Lower** | **Upper** | |  |
| **Cost type** | | | | | |
| Electrical Cardioversion | €965 | €581 | €1,331 | | (3) |
| ECV early postponed | €13.40 | €8.13 | €18.61 | | Assumption, (4) |
| ECV late postponed | €450 | €252 | €649 | | Assumption |
| ECV postponed for SSR | €422 | €250 | €593 | | Assumption, (3) |
| Rivaroxaban (week) | €16.99 |  |  | | (5) |
| VKA (week) | €0.40 |  |  | | (5) |
| Rate control  (metoprolol:digoxin, 50:50 [week]) | €1.84 |  |  | | (5) |
| INR monitoring pre-CV (week)  (on average 5.5 measurements) | €30.65 | €18.64 | €42.67 | | (6,7) |
| INR monitoring normal (week)  (home-monitoring vs thrombosis service: 43.1% vs 56.9%) | €6.52 | €3.96 | €9.08 | | (6) |
|  |  |  |  | |  |
| Ischemic Stroke | €17,745 | €10,789 | €24,700 | | (8) |
| Intracranial Hemorrhage | €14,119 | €8,584 | €19,654 | | (8) |
| Myocardial Infarction | €2,976 | €1,810 | €4,143 | | (9) |
| Major Hemorrhage | €10,497 | €6,382 | €14,611 | | (10) |
| Gastrointestinal Hemorrhage | €10,497 | €6,382 | €14,611 | | (10) |
| Minor Hemorrhage | €291 | €177 | €406 | | (4,5) |
|  |  |  |  | |  |
| Productivity loss (week) | €181 |  |  | | (11) |
| Productivity loss FCM 22 days | €798 |  |  | | (11) |
| Productivity loss FCM 30 days | €1,088 |  |  | | (11) |
| Productivity loss symptomatic AF FCM^*^ | €968 |  |  | | (5) |
| Productivity loss major event^†^ | €2,177 |  |  | | Assumption, (11) |
| Productivity loss hemorrhagic event^‡^ | €363 |  |  | | Assumption, (11) |
|  | | | | | |
| Informal care AF (week) | €112 |  |  | | Assumption, (12) |
| Informal care major event^†^ | €364 |  |  | | Assumption, (12) |
| Informal care bleeding event^‡^ | €224 |  |  | | Assumption, (12) |
|  |  |  |  | |  |
|  | **Mean estimate** | **95% CI estimate** | | |  |
|  |  | **Lower** | **Upper** | |  |
| **Utilities** | | | | | |
| Ischemic Stroke | 0.602 | 0.544 | 0.662 | | (8) |
| Intracranial Hemorrhage | 0.602 | 0.543 | 0.660 | | (8) |
| Myocardial Infarction | 0.680 | 0.604 | 0.756 | | (13) |
| Major Hemorrhage | 0.710 | 0.628 | 0.793 | | (13) |
| Gastro-Intestinal Hemorrhage | 0.710 | 0.628 | 0.793 | | (13) |
| Symptomatic AF | 0.699 | 0.620 | 0.776 | | (14) |
| Asymptomatic AF | 0.819 | 0.769 | 0.936 | | (14) |
| Permanent AF | 0.734 | 0.688 | 0.838 | | (14) |

Abbreviations: AF, Atrial Fibrillation; ECV, Electrical cardioversion; FCM, Friction Cost Method; INR, International Normalized Ratio; SSR, Spontaneous Sinus Rhythm; VKA, Vitamin K oral antagonist anticoagulant.

^*^ The friction period used is 12 weeks.

^†^ Major events are ischemic stroke, intracranial hemorrhage and myocardial infarction.

^‡^ Hemorrhagic events are gastrointestinal hemorrhages and major hemorrhages.

**Utility calculations**

The utilities for ischemic stroke (IS) and intracranial hemorrhage (ICH) were based on a non-randomized controlled cluster-trial which explored the medical costs concerning stroke services. Quality of life for IS and ICH were measured in this trial at hospital discharge and 6 months after the event occurred, subdivided based on modified Rankin Scales (mRS) of 0-1, 2-3, 4 and 5 (8). The utilities were recalculated into a single weighted average value that was applied to the acute event phase as well as the post event phase. The first 6 months were classified as the acute phase, the following 6 months were designated as the post event phase. An event was considered minor when the mRS score was 0-3, a major event was considered with a mRS score of 4-5.

Table S2. Distribution of severity of stroke and intracranial hemorrhage across modified Rankin Scales classes

|  | After 6 months | After 12 months |
| --- | --- | --- |
| mRS 0-3 | 60% | 71% |
| mRS 4-5 | 40% | 29% |

The same principle of the acute and post event phase also applied to the calculation of the MI utility. The acute phase for MI was considered the first 6 months, the post event phase had an utility that equaled the utility of symptomatic atrial fibrillation (AF)

Table S3. Distribution of severity of stroke and intracranial hemorrhage across modified Rankin Scales classes and associated costs recalculated into total event costs.

|  | mRS 0-1 | mRS 2-3 | mRS 4 | mRS 5 | Weighted total  Differentiated | Overall weighted total |
| --- | --- | --- | --- | --- | --- | --- |
| Percentage after 6 months | 16% | 44% | 32% | 8% |  |  |
| Percentage after 12 months | 24% | 47% | 26% | 3% |  |  |
| Costs 0-6 months | €9,856  ($12,228) | €14,868  ($18,446) | €37,628  ($46,682) | €46,089  ($57,179) |  |  |
| Costs 7-12 months | €1,761  ($2,185) | €4,196  ($5,206) | €17,824  ($22,113) | €22,515  ($27,933) |  |  |
|  |  |  |  |  |  |  |
| Weighted average (by %)  0-6 months | €1,577  ($1,956) | €6,542  ($8,116) | €12,041  ($14,938) | €3,687  ($4,574) | €23,847  ($29,585) | €17,744  ($22,014) |
| Weighted average (by %)  7-12 months | €423  ($525) | €1,972  ($2,447) | €4,634  ($5,749) | €675  ($837) | €7,704  ($9,558) |  |

**Non-healthcare costs**

Productivity loss calculation

Productivity loss was calculated into a single value based on the average population age of 64 years (standard deviation of 10.8 years), the ratio men : women, the labor market participation per 5-year age group, the average hours of work per week per 5-year age category and the average monthly gross salary per 5-year age category (11).

**Fig S1** Age distribution based on an average age of 64 years with a standard deviation of 10.8 years as found in the X-VeRT trial. A normal distribution of age was assumed

The gross hourly earnings were not available for people <45 year and >75 years. The gross hourly earnings for patients younger than 45 years were assumed to be equal to that of patients aged 45-50 years (see Table S4). Patients >75 years were excluded in the calculation of productivity loss since their labor market participation is <1%.The average age was used to compute the proportion of patients within the 5-year age groups ranging from 40 years up to 75 years in the base-case. The categorization in these age groups was chosen to align with the available economic data. The computed proportion per age group was used to calculate an overall weighted average hourly gross productivity loss in euros (€). All patients were assumed to have full loss of productivity while waiting for their electrical cardioversion (ECV) procedure. Patients with recurrent AF after the ECV procedure all had a productivity loss with a friction period equaling the average days up to a new ECV, i.e. 30 days for VKA and 22 days for rivaroxaban. Productivity loss in patients with permanent AF was only applied to patients categorized in modified European Heart Rhythm Association (mEHRA) class 2b, 3 and 4 using the friction cost method with a friction period of 12 weeks according to Dutch cost-effectiveness guidelines (4,14). Productivity loss for the major events ischemic stroke (IS), intracranial hemorrhage (ICH) and myocardial infarction (MI) was calculated with a 12 week friction period. Productivity loss for major hemorrhage (MaH) and gastrointestinal hemorrhage (GIH) was calculated with a 2 week friction period, no productivity loss was assumed for minor hemorrhage (MiH).

Table S4: Variables used for productivity loss calculations.

| Age group | Labor force men (%, 2015) | Labor force women (%, 2015) | Gross hourly wage men  (2002)* | Gross hourly wage women  (2002)* | Hours’ work /week, men (2015) | Hours’ work /week, women (2015) |
| --- | --- | --- | --- | --- | --- | --- |
| 40-45 years | 88.3% | 75.8% | €21.86  ($26.90) | €16.80  ($20.84) |  |  |
| 45- 50 years | 88.2% | 75.9% |  |  | 40 | 26 |
| 50- 55 years | 84.6% | 71.0% |  |  | 40 | 26 |
| 55-60 years | 79.3% | 61.1% |  |  | 37 | 25 |
| 60- 65 years | 57.7% | 35.3% |  |  | 37 | 25 |
| 65- 70 years | 13.6% | 4.0% |  |  | 21 | 14 |
| 70- 75 years | 4.9% | 2.4% |  |  | 21 | 14 |

* Weighted average based on highest education achieved and all jobs weight by labor market participation. The last representative calculation that could be derived from Statline was from 2002. Statline is a web interface of the Statistics Netherlands (CBS).

Informal care costs

The costs of informal care were subdivided into ‘non-intensive’ with 8 hours of care provided per week and ‘intensive’ with on average 26 hours of care provided per week (12). The ‘non-intensive’ informal care was considered for symptomatic AF, permanent AF and patients who experienced a MaH or GIH. The ‘intensive’ informal care was considered for the major events IS, MI and ICH during the remaining time horizon. The indirect costs, both productivity loss and informal care, were applied to all symptomatic patients prior to ECV or recurrent patients with a mEHRA classification of 3 or 4 (70%) and to the patients with permanent AF mEHRA class 3 and 4 (32%) during the remaining time horizon (14).

**References**

(1) Van Gelder IC, Crijns HJ, Tieleman RG, Brugemann J, De Kam PJ, Gosselink AT et al. Chronic atrial fibrillation. Success of serial cardioversion therapy and safety of oral anticoagulation. Arch Intern Med 1996;156(22):2585-2592.

(2) Camm AJ, Amarenco P, Haas S, Hess S, Kirchhof P, Kuhls S et al. XANTUS: a real-world, prospective, observational study of patients treated with rivaroxaban for stroke prevention in atrial fibrillation. Eur Heart J 2016;37(14):1145-1153.

(3) Nederlandse Healthcare Authority (NZa). NZa Zorgproducten Tariefapplicatie. 2017; Available at: http://dbc-zorgproducten-tarieven.nza.nl. Accessed March 21, 2017.

(4) Dutch Health Institute. Kostenhandleiding: Methodologie van kostenonderzoek en referentieprijzen voor economische evaluaties in de gezondheidszorg. 2015; Available at: www.zorginstituutnederland.nl. Accessed January 6, 2017.

(5) Dutch Healthare Institute. The Pharmacy Purchase Price. 2016; Available at: https://www.medicijnkosten.nl/. Accessed January 11, 2016.

(6) Federatie van Nederlandse Trombosediensten (FNT). Samenvatting medische jaarverslagen 2014.

(7) Federatie van Nederlandse Trombosediensten (FNT). De kunst van het doseren. 2014;3e druk.

(8) Baeten SA, van Exel NJ, Dirks M, Koopmanschap MA, Dippel DW, Niessen LW. Lifetime health effects and medical costs of integrated stroke services - a non-randomized controlled cluster-trial based life table approach. Cost Eff Resour Alloc 2010;8:21-7547-8-21.

(9) Soekhlal RR, Burgers LT, Redekop WK, Tan SS. Treatment costs of acute myocardial infarction in the Netherlands. Neth Heart J 2013;21(5):230-235.

(10) Vonkeman HE, Klok RM, Postma MJ, Brouwers JR, van de Laar MA. Direct medical costs of serious gastrointestinal ulcers among users of NSAIDs. Drugs Aging 2007;24(8):681-690.

(11) Statistics Netherlands (CBS). Statline. 2015; Available at: http://statline.cbs.nl/Statweb/. Accessed January 17, 2017.

(12) De Klerk M, De Boer A, Plaisier I, Schyns P, Kooiker S. Informele hulp: wie doet er wat? 2015;2015-35.

(13) Sullivan PW, Slejko JF, Sculpher MJ, Ghushchyan V. Catalogue of EQ-5D scores for the United Kingdom. Med Decis Making 2011;31(6):800-804.

(14) Wynn GJ, Todd DM, Webber M, Bonnett L, McShane J, Kirchhof P et al. The European Heart Rhythm Association symptom classification for atrial fibrillation: validation and improvement through a simple modification. Europace 2014;16(7):965-972.
